# Expression profiling in spondyloarthropathy synovial biopsies highlights changes in expression of inflammatory genes in conjunction with tissue remodelling genes

**DOI:** 10.1186/1471-2474-14-354

**Published:** 2013-12-15

**Authors:** Gethin P Thomas, Ran Duan, Allison R Pettit, Helen Weedon, Simranpreet Kaur, Malcolm Smith, Matthew A Brown

**Affiliations:** 1 The University of Queensland Diamantina Institute, Translational Research Institute, 37 Kent St, Woolloongabba, QLD 4102, Australia; 2 The University of Queensland Diamantina Institute, Princess Alexandra Hospital, Brisbane, Australia; 3 Mater Research, Translational Research Institute, 37 Kent St, Woolloongabba, QLD 4102, Australia; 4 Australia and University of Queensland Centre for Clinical Research, Royal Brisbane & Women’s Hospital Campus, Bowen Bridge Road, Herston, QLD 4029, Australia; 5 Repatriation General Hospital, Daw Park, SA 5041, Australia; 6 The University of Queensland Centre for Clinical Research, Brisbane, QLD 4029, Australia

**Keywords:** Ankylosing spondylitis, Synovial membrane, Spondyloarthritis, Inflammation, MMP3, Gene expression, Microarrays

## Abstract

**Background:**

In the spondyloarthropathies, the underlying molecular and cellular pathways driving disease are poorly understood. By undertaking a study in knee synovial biopsies from spondyloarthropathy (SpA) and ankylosing spondylitis (AS) patients we aimed to elucidate dysregulated genes and pathways.

**Methods:**

RNA was extracted from six SpA, two AS, three osteoarthritis (OA) and four normal control knee synovial biopsies. Whole genome expression profiling was undertaken using the Illumina DASL system, which assays 24000 cDNA probes. Differentially expressed candidate genes were then validated using quantitative PCR and immunohistochemistry.

**Results:**

Four hundred and sixteen differentially expressed genes were identified that clearly delineated between AS/SpA and control groups. Pathway analysis showed altered gene-expression in oxidoreductase activity, B-cell associated, matrix catabolic, and metabolic pathways. Altered "myogene" profiling was also identified. The inflammatory mediator, *MMP3*, was strongly upregulated (5-fold) in AS/SpA samples and the Wnt pathway inhibitors *DKK3* (2.7-fold) and *Kremen1* (1.5-fold) were downregulated.

**Conclusions:**

Altered expression profiling in SpA and AS samples demonstrates that disease pathogenesis is associated with both systemic inflammation as well as local tissue alterations that may underlie tissue damaging modelling and remodelling outcomes. This supports the hypothesis that initial systemic inflammation in spondyloarthropathies transfers to and persists in the local joint environment, and might subsequently mediate changes in genes directly involved in the destructive tissue remodelling.

## Background

The underlying processes driving disease progression in the spondyloarthropathies (SpA) are very poorly understood. The disease transitions from an initial inflammatory insult through an inflammation-driven tissue destruction phase to an osteoproliferative phase which in the worst cases results in joint fusion. SpAs mainly present in the axial skeleton and the inaccessibility of these joints and subsequent lack of sample availability together with the slow disease progression hinders research such that the dysregulated molecular and cellular mechanisms driving disease remain largely unknown.

Expression profiling studies of affected tissues in SpA offer a hypothesis free approach to elucidating underlying pathogenic mechanisms. Previously ours and other groups have focussed largely on peripheral blood samples, either from whole blood [1,2] or from total [3,4] or partial [5] PBMC fractions. These studies provide valuable information regarding the systemic immunological processes involved in SpA, they are less informative regarding local inflammatory and tissue damage processes, in particularly the mechanisms underlying joint damage and the progression from inflammation to osteoproliferation in SpA.

Until very recently, only two small-scale tissue expression profiling studies have been undertaken in SpA, in synovial biopsies [6] and sacroiliac joint fluid [7], and no comprehensive genomic profiling study had been reported in joint tissue in SpA.

Peripheral arthritis is present in significant numbers of SpA patients with estimates between 14-20% of AS patients and 18-26% of Undifferentiated SpA patients [8]. In ankylosing spondyltitis (AS) patients with both axial and peripheral inflammation, anti-TNF treatments, such as adalimumab, have shown efficacy in reducing both peripheral and axial disease [9]. This site inclusive treatment efficacy suggests similar disease processes are occurring in these different joint environments. Subsequently this provides some justification for assessment of molecular changes within affected knee joints, that are a more accessible tissue site, as a viable approach for elucidating joint specific disease processes in SpA.

In early 2013, Yeremenko *et al.* published a study in which they undertook a large-scale gene expression profiling study comparing knee synovial biopsies from SpA, rheumatoid arthritis (RA) and gout patients. They demonstrated that many inflammatory genes and pathways were shared across RA and SpA. However, a "myogenic" profile was evident in the SpA samples which delineated them from the RA samples [10].

We have undertaken a similar approach, comparing archived formaldehyde-fixed paraffin-embedded (FFPE) synovial biopsies from AS, SpA, normal control and osteoarthritis (OA) patients. We similarly identified an enhanced myogene signature in our AS/SpA samples. Additionally we have also identified a number of other pathways that may contribute to tissue remodelling as well as inflammatory pathways.

## Method

### Patients

Fifteen knee synovial biopsy tissue samples consisting of six seronegative spondyloarthropathy (SpA), two ankylosing spondylitis (AS), three osteoarthritis (OA) and four normal control biopsies were obtained from the Synovial Tissue Bank at the Repatriation General Hospital in Adelaide, South Australia (Additional file 1: Table S1). Biopsies were taken arthroscopically under direct vision biopsying with sampling of macroscopically abnormal appearing synovium. All patients provided informed written consent. Ethical approval for this study was obtained from the Southern Adelaide Health Service/Flinders University Human Research Ethics Committee.

### RNA preparation and Microarray analysis

RNA was extracted from the biopsies embedded in formaldehyde-fixed paraffin-embedded (FFPE) tissue blocks using the Arcturus Paradise^©^ Plus Reagent System (Molecular Devices, Sunnyvale, CA) as per the manufacturer’s protocol. All the biopsy was used for the RNA extraction.

200 ng of RNA was used in the Illumina Whole-Genome DASL® (cDNA-mediated Annealing, Selection, Extension, and Ligation) Gene Expression Assay according to the Illumina protocol. This technique has been specifically developed for whole-genome expression profiling of degraded RNA samples from archived tissue biopsies. RNA is first converted to cDNA through a reverse transcription reaction with biotinylated primers. The biotinylated cDNA is then annealed to assay oligonucleotide probes specific for each of the 24000 cDNAs targeted by the array. The hybridized oligonucleotides are then extended and ligated in a second-strand cDNA synthesis forming a synthetic template that is transferred to a PCR reaction containing a fluorescently labelled primer. The labelled PCR product strand is then isolated and the fluorescent products were hybridised to Illumina Ref-8 Expression BeadChips and scanned. Gene expression is then quantified by the level of fluorescent hybridization to the BeadChip. Data was processed in GenomeStudio (Illumina) and analysed using Lumi [11] and BRB ArrayTools [12] as described previously [3]. Data was transformed by variance stabilization transformation (VST) [13] then normalized by robust spline normalization (RSN) [14]. This data has been uploaded to the NCBI GEO database and assigned accession number GSE41038.

Of the 24,500 cDNAs on the DASL arrays, 20,700 were found to be expressed in at least one sample and were included in the analysis. For analysis, AS and SpA samples were grouped together and compared with a control group consisting of OA and normal samples. Differentially expressed genes were identified by unpaired *t*-test with multivariate permutation correction. The evaluation of which Gene Ontology (GO) classes are differentially expressed between control and affected bones was performed using a functional class scoring analysis as described previously [2]. Efron-Tibshirani’s Gene Set Analysis (GSA) was used which uses "maxmean" statistics for assessing significance of pre-defined gene-sets. Gene Ontology analysis was performed using BRB-ArrayTools.

### Quantitative PCR

Quantitative PCR validation (qPCR) was carried out as described previously [3] in nine normal and OA samples as well as in seven SpA and AS samples. Due to low RNA yields obtained from the biopsies four of the array samples lacked sufficient RNA for confirmation qPCR follow-up but an additional five control samples were obtained for the qPCR analysis generating a partially independent confirmation cohort (Additional file 1: Table S1).

Briefly, cDNA was generated from 1 μg of total RNA using the Bioline cDNA synthesis Kit (Bioline, London, UK) according to manufacturer’s instructions. Candidate genes were assayed using the predesigned TaqMan assays (DKK3 = Hs00247426_m1, PTGER4 = Hs00168761_m1, MMP3 = Hs00968308_m1). For normalisation, expression levels of the housekeeping gene, RPL32, [8] were measured by SYBR green based qRT-PCR using specific forward (5′-CCCCTTGTGAAGCCCAAGA-3′) and reverse (5′-GACTGGTGCCGGATGAACTT-3′) primers. All assays were carried out using SensiMix dT RT-PCR reagent (Quantace, Sydney, Australia) under the following conditions; 50°C for 2 min, 95°C for 10 min, and 40 cycles of 95°C for 15 s and 60°C for 60s.

Relative expression of genes of interest were determined using the ΔCT method or standard curve method. Comparisons between different patient groups were undertaken using Mann–Whitney tests.

### Immunohistochemistry

For the MMP3 immunohistochemistry, three AS, five SpA, 9 normal and 24 RA biopsies were stained. Tissue sections were blocked for endogenous peroxidase before digestion with proteinase K. This was followed by incubation first with a mouse anti-human MMP3 primary antibody (Santa Cruz Biotechnology, Santa Cruz, CA) for 2 hrs at room temperature (RT) then with a donkey anti-mouse IgG secondary antibody (Jackson ImmunoResearch Labs, West Grove, PA) for 40 mins at RT. Antibody staining was visualised with an ABC kit (Vector Laboratories, Burlingame, CA) using an AEC chromagen substrate (Dako, Cambellfield, Australia) before counterstaining with haematoxylin and mounting with Aquatek (Merck, Kilsyth, Australia). Staining was quantified using NIS Elements Br 3.0 software (Nikon, Lidcombe, Australia).

## Results

**Figure 1:**
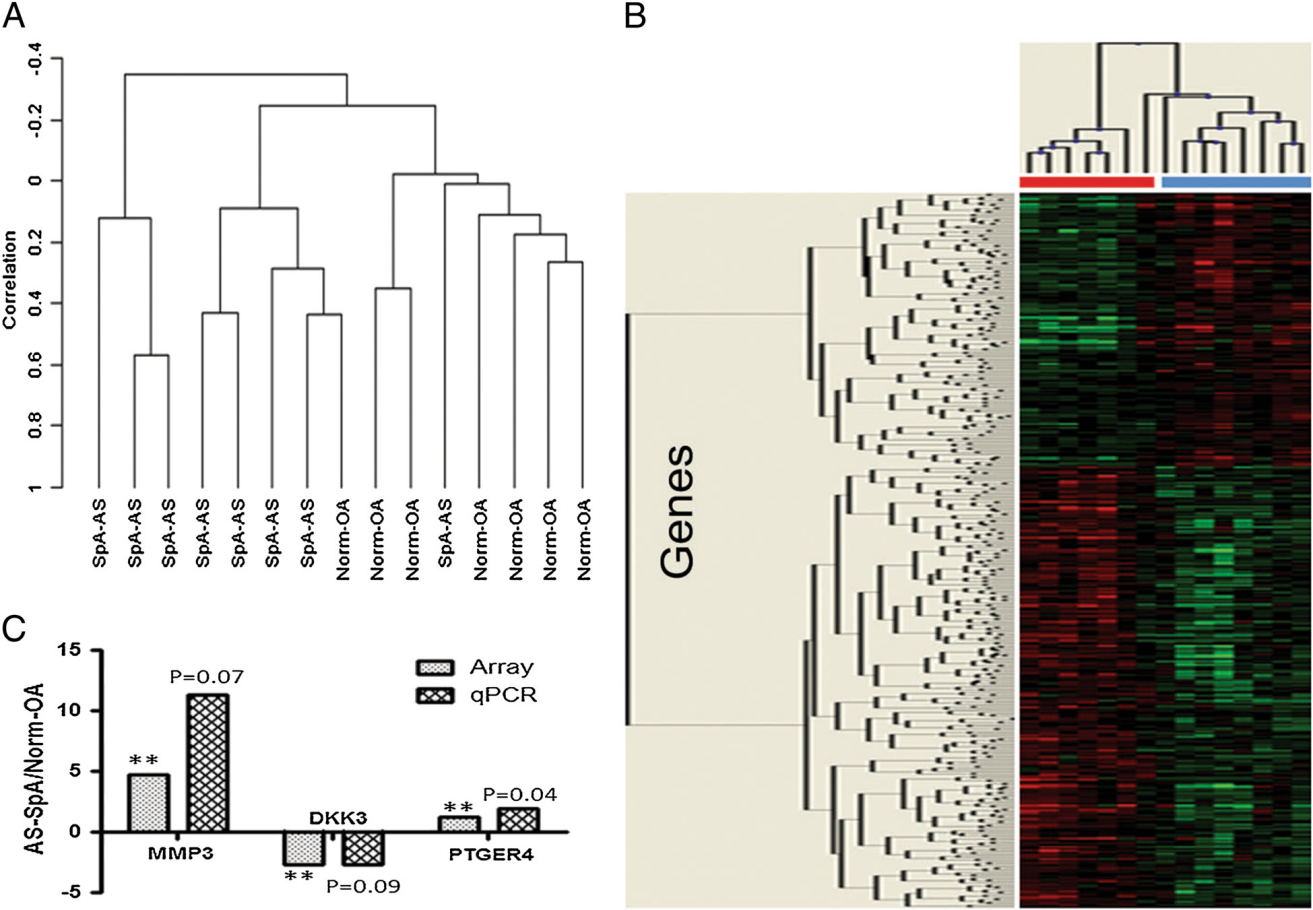
**Histological and immunohistochemical findings. (A)** Diffuse lymphocytic infiltration of the dermis predominantly composed of large pleomorphic cells with irregular nuclei and prominent nucleoli (hematoxylin-eosin staining, magnification 20×). By immunoperoxidase staining, the neoplastic cells showed marked and diffuse expression of CD3 **(B)** and CD30 **(C)**.

To maximise the power of the study we grouped the eight AS and SpA samples together (AS-SpA) and compared them with a control group consisting of seven normal and OA (a non-inflammatory arthritis) (OA-Norm) samples for the analysis. The validity of this grouping was confirmed by unsupervised clustering that showed no differences between AS and SpA nor between OA and normal samples (data not shown). However, unsupervised clustering clearly delineated between the AS-SpA and OA-Normal groups, with only one sample from each group misclassifying (Figure 1A, sensitivity 88%, specificity 86%).

To identify differentially expressed genes we undertook a class comparison of the two groups which showed this clustering was driven by 416 differentially expressed genes (p < 0.01) ranging from a 4.7-fold up-regulation to a 4.6-fold down-regulation (Figure 1B, Additional file 2: Table S2).

To ascertain if there was a correlation in the tissues with systemic inflammatory genes dysregulated in our previous PBMC expression profiling studies [3] we compared the genelists. Using Gene-set Enrichment Analysis (GSEA) to calculate the degree of enrichment of the synovial biopsy genelist in the transcriptome of the AS PBMCs, Efron-Tibshirani’s GSA maxmean test showed the synovial geneset was enriched in the PBMC transcriptome with a p-value of 0.005. A number of immune/inflammation-associated genes were altered in the two datasets (highlighted in Additional file 2: Table S2). The upregulated genes were *CD40* (a member of the TNF receptor superfamily); *CLEC12A* (a member of the C-type lectin/C-type lectin-like domain superfamily); and *FCGR1A* (a high-affinity Fc-gamma receptor). Conversely, *TSC22D3*, which plays a key role in the anti-inflammatory and immunosuppressive effects of glucocorticoids, was downregulated in both PBMCs and synovial biopsies.

To identify changes in pathways that might mediate disease we undertook Gene Ontology (GO) analysis. In the synovial biopsies, a number of inflammatory pathways showed altered expression including those involving oxidoreductase activity (including the cyclooxygenases which mediate prostaglandin production), B-cell activity, interferon-γ response and myeloid cell activation (Table 1).

**Table 1:** Gene ontology analysis of differentially expressed genes

**Gene ontology term**	**GO category**	**Efron-Tibshirani’s GSA test p-value**
**Collagen/extracellular matrix association**		
Collagen binding	GO:0005518	< 0.005
Collagen metabolic process	GO:0032963	0.05
Collagen catabolic process	GO:0030574	0.035
Integrin complex	GO:0008305	< 0.005
Substrate-bound cell migration	GO:0006929	< 0.005
Negative regulation of cytoskeleton organization	GO:0051494	0.025
**Nitric oxide/monooxygenase activity**		
Regulation of monooxygenase activity	GO:0032768	< 0.005
Regulation of oxidoreductase activity	GO:0051341	< 0.005
Regulation of nitric-oxide synthase activity	GO:0050999	0.005
Nitric oxide biosynthetic process	GO:0006809	0.015
Positive regulation of monooxygenase activity	GO:0032770	0.005
Nitric oxide metabolic process	GO:0046209	0.01
Regulation of nitric oxide biosynthetic process	GO:0045428	0.005
Regulation of calcidiol 1-monooxygenase activity	GO:0060558	0.005
**Immune associated functions**		
B cell receptor signaling pathway	GO:0050853	0.015
Regulation of leukocyte mediated immunity	GO:0002703	0.02
Response to interferon-gamma	GO:0034341	0.005
Positive regulation of B cell activation	GO:0050871	0.04
Humoral immune response mediated by circulating immunoglobulin	GO:0002455	0.02
Myeloid cell activation during immune response	GO:0002275	0.01
Regulation of lymphocyte mediated immunity	GO:0002706	0.035
**Muscle/myocyte/myofibroblast biology**		
Dystroglycan binding	GO:0002162	0.015
Myosin complex	GO:0016459	0.01
Muscle filament sliding	GO:0030049	0.02
Actin-myosin filament sliding	GO:0033275	0.02
Myosin II complex	GO:0016460	0.025
Actomyosin structure organization	GO:0031032	0.04
Regulation of myoblast differentiation	GO:0045661	0.03
Muscle myosin complex	GO:0005859	0.02
Myoblast differentiation	GO:0045445	0.02
Regulation of myotube differentiation	GO:0010830	0.015
Myotube differentiation	GO:0014902	0.005

Gene ontology (GO) analysis was undertaken using BRB Array Tools. Significantly altered genesets were identified using Efron-Tibshirani’s Gene Set Analysis (GSA) test which tests which genesets contain more differentially expressed genes than would be expected by chance. The threshold of determining significant gene sets was 0.005.

**Figure 2:**
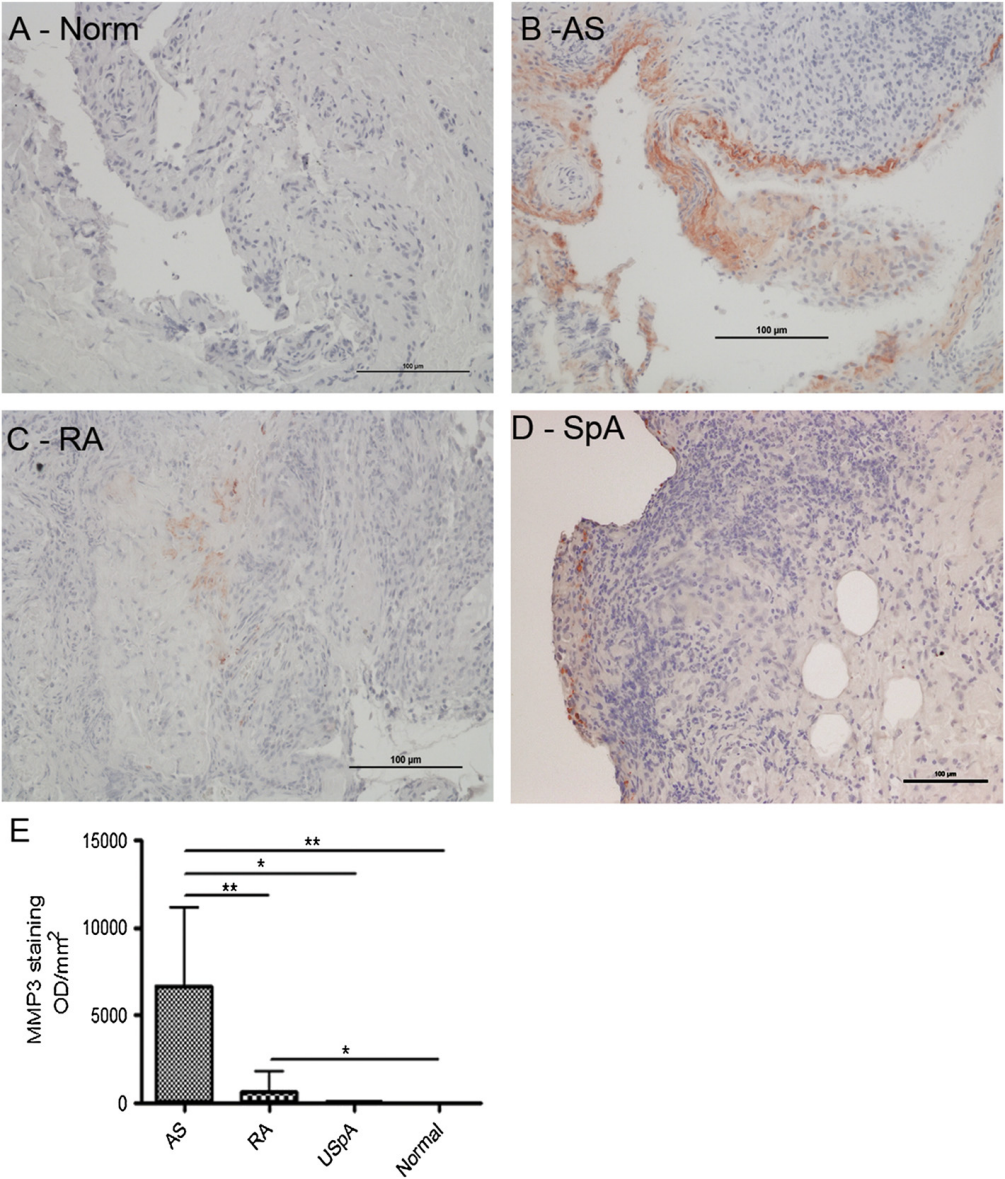
**Immunohistochemistry showing MMP3 protein levels in synovial biopsies.** MMP3 was undetectable in normal biopsies **(A)** with very low levels seen in RA **(C)** with similarly low levels in SpA **(D)** samples and high expression of MMP3 in AS samples **(B)**. Quantitation of the stain is shown in **(E)**.

We also specifically focused on gene expression changes that might contribute directly to the tissue remodelling seen in affected joints in SpA. The tissue remodelling inflammatory genes, *matrix metalloproteinase 1* (*MMP1*, 3.5-fold, p = 0.001) and *matrix metalloproteinase 3* (*MMP3*, 4.7-fold, p = 0.005) showed marked up-regulation in AS/SpA biopsies (Table 2). Quantitative PCR confirmed these changes showing an 11-fold upregulation in MMP-3 expression (Figure 1C). Robust MMP-3 protein expression was detected by immunohistochemistry in AS biopsies (6700 OD/mm2) with low expression in SpA (32 OD/mm2) and RA (652 OD/mm2) samples. MMP-3 protein expression was not detected in normal control samples (Figure 2). MMP-3 RNA levels were also higher in the two AS samples than in the SpA samples, though not significantly. The prostaglandin E receptor 4 (*PTGER4)* was also upregulated (1.24-fold by microarray, 1.9-fold by qPCR, p < 0.05). Gene ontology analysis identified matrix catabolic and metabolic pathway dysregulation (Figure 1).

**Table 2:** Expression levels of candidate genes on the microarray and in the qPCR confirmation study

**Array data**	**Norm-OA (9)**		**AS-SpA (6)**		**AS-SpA/Norm-OA**	**p-value**
MMP3	480.66		2260.32		4.7	0.005255
MMP1	199.47		701.47		3.51	0.001053
DKK3	517.49		190.98		-2.71	0.003263
KREMEN1	988.68		674.73		-1.46	6.50E-03
PTGER4	317.58		393.08		1.24	4.70E-02
**qPCR**	**Norm-OA (9)**	**SD**	**AS-SpA (7)**	**SD**	**AS-SpA/Norm-OA**	**p-value**
MMP3	0.00026	0.00058	0.00295	0.00412	11.35	0.07
DKK3	0.101	0.073	0.037	0.061	-2.7	0.086
PTGER4	66.6	62.9	126.7	50.1	1.9	0.042

Two Wnt pathway inhibitory genes were down-regulated in our microarray dataset (Figure 1C, Table 2); *DKK3* (2.7-fold, p = 0.003) and *Kremen1* (1.5-fold, p = 0.007). Quantitative PCR data supported the array findings with *DKK3* down-regulated 2.7-fold (p = 0.09, Figure 1C, Table 2); *DKK3* was in fact undetectable in the AS samples with low levels of expression in the SpA samples (data not shown).

A recent study demonstrated a strong enhancement of a "myogene signature" in AS and SpA synovial biopsies [10]. We also saw alterations in a number of myocyte-associated pathways (Table 1). However when we looked specifically at the genes differentially expressed in the myogene signature in the Yeremenko study we did see not strong expression changes suggesting our myogene signature was due to a different subset of genes (data not shown).

## Discussion and conclusions

Using whole genome expression profiling in archived synovial biopsies we have established changes in key pathways and genes that might mediate both the inflammatory changes and the tissue remodelling downstream of the inflammation in SpA and AS.

Estimates of the incidence of peripheral arthritis are between 20-50% in AS and SpA patients [8,15,16]. It has been proposed that the aetiopathogenesis of peripheral and axial SpA are similar [8,17]. In both cases inflammation arises close to the enthesis with the inflammatory infiltrate sharing many common features at the two sites [17]. Whether enthesitis is the underlying initiating pathology driving disease in SpA is still a subject of considerable debate [18,19].

As might be expected in inflammatory arthritidies such as SpA and AS, immune pathways are affected. Comparison of this synovial tissue dataset with our previously published PBMC dataset [3] identified a subset of inflammatory genes and pathways that were altered in both studies. Similar dysregulation in the interferon response and myeloid cell pathways was seen possibly reflecting systemic changes. Localised tissue inflammatory pathways such as the oxidoreductase pathways however are altered in synovial tissue but not PBMCs. Differentially regulated pathways potentially mediate the progression from systemic inflammation to localised inflammatory-driven tissue damage.

In synovium, a number of closely-associated inflammatory pathways involved in oxidoreductase activity were identified, which includes the monooxygenase pathways involved in nitric oxide production, and the cyclooxygenase pathways producing COX-1 and COX-2 produce prostanoids such as prostaglandins. COX-2 expression has previously been demonstrated in SpA-affected joints [20]. Cyclo-oxygenase inhibition using non-steroidal anti-inflammatory drugs is a mainstay of therapy in AS, and there is even suggestive evidence that such treatment may retard the progression of ankylosis in the disease [21-23]. Prostaglandin E receptor 4 (*PTGER4*) was also upregulated, which has been shown to be associated with AS in genomewide association studies [24]. This is of particular interest, as PTGER4 through its ligand PGE_2_ is a good molecular candidate to link physical stress at entheses with bone formation [25], and in driving inflammation through stimulation of IL-23 production by dendritic cells [26].

Further alterations at the tissue level were seen in pathways affecting collagen metabolism and catabolism, cell-motility and extracellular matrix interactions reflecting the inflammatory joint destruction and tissue remodelling seen in SpA. These were not altered in our studies on whole blood and PBMCs [2,3].

MMP-3 was one of the most strongly upregulated genes. Members of the MMP family of stromelysins have been well documented to play roles in inflammation-mediated tissue destruction. Elevated serum levels of MMP-3 have been indicated in AS as a systemic biomarker of disease progression and activity [27], and correlate well with BASDAI [28] and response to TNF-blockade treatment [29,30]. In a study on SpA patients with peripheral joint involvement, high serum MMP-3 correlated closely with increased synovial fibroblast MMP-3 production supporting a local joint source for the serum levels. MMP3 levels have been suggested to be the best predictor of peripheral arthritis treatment response [31]. In fact high MMP3 production was proposed as a diagnostic biomarker for peripheral involvement rather than global inflammation in SpA. High serum MMP3 levels (presumably originating from the synovitis) differentiated those patients suffering from axial and peripheral SpA from those with only axial SpA [31]. Even though synovial inflammation in RA is usually more destructive than that in SpA, MMP3 levels are still higher in SpA suggesting a different tissue remodelling role for MMP3 in SpA.

The Wnt pathway has been identified as playing an important role in mediating bone formation and release of inhibition of this pathway has been suggested to contribute to osteoproliferation both in AS [32] and in mouse models of SpA [33]. Downregulation of Wnt inhibitors, such as *DKK3* and *Kremen1,* as suggested by the current data, could therefore generate permissive signals for the excess bone formation seen in AS. Osteoproliferation/bone formation in the synovial joints of SpA patients has not been described however, though bone formation in the affected entheses of SpA patients has been demonstrated [18,19].

In a similar study to this one, Yerenmenko *et al.* undertook a large scale whole genome expression profiling study comparing SpA with RA and gout synovial biopsies rather than OA and normal samples [10]. The key finding from this study was the identification of a 296-gene "myogene" expression profile that was highly enriched for genes associated with muscle/myocyte/myofibroblast biology. Interestingly, they did not report strong upregulation of inflammatory genes possibly due to the comparison being between two inflammatory arthritidies, although MMP1 was upregulated in the SpA samples. They also reported altered expression of genes in the Wnt pathway.

Similarly we also saw changes in "myogene" associated pathways, further supporting their proposal for fibrotic changes in the synovium of SpA patients. The specific gene changes underlying these pathways were not the same in the two studies but this may reflect the different patient cohorts and tissue processing (FFPE vs. fresh frozen). Analysis of our previous expression profiling studies in PBMCs and whole blood showed the absence of a myogene signature in these datasets suggesting it is a disease-site specific phenomenon [2,3]. Interestingly, gene ontology analysis comparing expression profiling of spines and knees in proteoglycan induced spondylitis (PGISp) mice showed a greater number of muscle-associated pathways upregulated in the knee joints suggesting this may be a unique feature of peripheral disease [33]. The significance of the myogene profile though remains to be elucidated however.

Two samples (1 OS-Normal and 1 AS-SpA) misclassified during the clustering analysis. There were no technical issues identified that might underline this so we can assume the reasons were biological. The misclassification of the sample probably reflects the compounded biological variation in SpA patients due to a combination of genetic factors and disease heterogeneity reflecting onset, severity and symptoms.

Although we identified some key pathways and genes of interest in this study it must e regarded as an exploratory study at this time. Despite some of the findings agreeing with previous studies [10], further independent validation studies are required to confirm the significance of our initial findings.

By adopting a whole genome profiling approach this study has identified gene signatures differentiating SpA from non-SpA samples and highlighting pathways that might play key pathophysiological roles in AS. Further, the candidate gene changes we have highlighted possible disease pathways that might control the progression through the inflammation and tissue destructive/osteoproliferative phases of spondyloarthropathy and provide guidance for focusing research efforts to elucidate disease mechanisms.

## Abbreviations

AS: Ankylosing spondylitis; PGISp: Proteoglycan-induced spondylitis; IHC: Immunohistochemistry; RA: Rheumatoid arthritis; IVD: Intervertebral disc; MRI: Magnetic resonance imaging; TNF: Tumour necrosis factor; HLA: Human leukocyte antigen; LRP: Low-density lipoprotein receptor-related protein; GSK: Glycogen synthase kinase; H&E: Haematoxylin and eosin; EDTA: Ethylenediaminetetraacetic acid; PG: Proteoglycan; RNA: Ribonucleic acid; MMP: Matrix metalloproteinase.

## Competing interests

The authors declare that they have no competing interests.

## Authors’ contributions

GPT conceived and designed study, analysed data and wrote manuscript. RD generated and analysed data and edited manuscript. ARP provided technical expertise, analysed data and wrote manuscript. HW generated and analysed data and edited manuscript. SK generated and analysed data and edited manuscript. MS provided clinical samples, expert opinion and edited the manuscript. MAB conceived study and wrote manuscript. All authors read and approved the final manuscript.

## Pre-publication history

The pre-publication history for this paper can be accessed here:

http://www.biomedcentral.com/1471-2474/14/354/prepub

## Supplementary Material

Additional file 1: Table S1 Clinical data for patients and controls. Click here for file

Additional file 2: Table S2 Class comparison of the Norm-OA and AS-SpA groups identified 416 differentially expressed genes (p < 0.01) ranging from a 4.7-fold up-regulation to a 4.6-fold down-regulation. Click here for file

## References

[B1] Sharma S Choi D Planck S Harrington C Austin C Lewis J Diebel T Martin T Smith J Rosenbaum J Insights in to the pathogenesis of axial spondyloarthropathy based on gene expression profiles Arthritis Res Ther 2009 14 6 R168 10.1186/ar2855 19900269 PMC3003511

[B2] Pimentel-Santos F Ligeiro D Matos M Mourao A Costa J Santos H Barcelos A Godinho F Pinto P Cruz M Whole blood transcriptional profiling in ankylosing spondylitis identifies novel candidate genes that might contribute to the inflammatory and tissue-destructive disease aspects Arthritis Res Ther 2011 14 2 R57 10.1186/ar3309 21470430 PMC3132052

[B3] Duan R Leo P Bradbury L Brown MA Thomas G Gene expression profiling reveals a downregulation in immune-associated genes in patients with AS Ann Rheum Dis 2010 14 1724 1729 10.1136/ard.2009.111690 19643760

[B4] Gu J Wei YL Wei JC Huang F Jan MS Centola M Frank MB Yu D Identification of RGS1 as a candidate biomarker for undifferentiated spondylarthritis by genome-wide expression profiling and real-time polymerase chain reaction Arthritis Rheum 2009 14 11 3269 3279 10.1002/art.24968 19877080 PMC2936922

[B5] Smith JA Barnes MD Hong D Delay ML Inman RD Colbert RA Gene expression analysis of macrophages derived from ankylosing spondylitis patients reveals interferon-gamma dysregulation Arthritis Rheum 2008 14 6 1640 1649 10.1002/art.23512 18512784 PMC2888278

[B6] Rihl M Baeten D Seta N Gu J De Keyser F Veys EM Kuipers JG Zeidler H Yu DTY Technical validation of cDNA based microarray as screening technique to identify candidate genes in synovial tissue biopsy specimens from patients with spondyloarthropathy Ann Rheum Dis 2004 14 5 498 507 10.1136/ard.2003.008052 15082479 PMC1755002

[B7] Rihl M Kellner H Kellner W Barthel C Yu DT Tak PP Zeidler H Baeten D Identification of interleukin-7 as a candidate disease mediator in spondylarthritis Arthritis Rheum 2008 14 11 3430 3435 10.1002/art.23998 18975340

[B8] Carron P Van Praet L Van den Bosch F Peripheral manifestations in spondyloarthritis: relevance for diagnosis, classification and follow-up Curr Opin Rheumatol 2012 14 4 370 374 10.1097/BOR.0b013e32835448de 22617824

[B9] Rudwaleit M Claudepierre P Kron M Kary S Wong R Kupper H Effectiveness of adalimumab in treating patients with ankylosing spondylitis associated with enthesitis and peripheral arthritis Arthritis Res Ther 2010 14 2 R43 10.1186/ar2953 20230622 PMC2888191

[B10] Yeremenko N Noordenbos T Cantaert T van Tok M van de Sande M Cañete JD Tak PP Baeten D Disease-specific and inflammation-independent stromal alterations in spondylarthritis synovitis Arthritis & Rheumatism 2013 14 1 174 185 10.1002/art.37704 22972410

[B11] Du P Kibbe WA Lin SM Lumi: a pipeline for processing Illumina microarray Bioinformatics 2008 14 13 1547 1548 10.1093/bioinformatics/btn224 18467348

[B12] Simon R Lam A Ming-Chung L Ngan M Menenzes S Zhao Y Analysis of Gene Expression Data Using BRB-Array Tools Cancer Inform 2007 14 11 17 19455231 PMC2675854

[B13] Lin SM Du P Huber W Kibbe WA Model-based variance-stabilizing transformation for Illumina microarray data Nucleic Acids Res 2008 14 2 e11 18178591 10.1093/nar/gkm1075 PMC2241869

[B14] Workman C Jensen LJ Jarmer H Berka R Gautier L Nielser HB Saxild HH Nielsen C Brunak S Knudsen S A new non-linear normalization method for reducing variability in DNA microarray experiments Genome Biol 2002 14 9 research0048 12225587 10.1186/gb-2002-3-9-research0048 PMC126873

[B15] Maksymowych WP Chou CT Russell AS Matching prevalence of peripheral arthritis and acute anterior uveitis in individuals with ankylosing spondylitis Ann Rheum Dis 1995 14 2 128 130 10.1136/ard.54.2.128 7702400 PMC1005535

[B16] Kristensen LE Karlsson JA Englund M Petersson IF Saxne T Geborek P Presence of peripheral arthritis and male sex predicting continuation of anti–tumor necrosis factor therapy in ankylosing spondylitis: an observational prospective cohort study from the south swedish arthritis treatment group register Arthritis Care Res 2010 14 10 1362 1369 10.1002/acr.20258 20506310

[B17] Vandooren B Tak P Baeten D López-Larrea C, Díaz-Peña R Synovial and Mucosal Immunopathology in Spondyloarthritis Molecular Mechanisms of Spondyloarthropathies 2009 vol. 649 New York: Springer 71 84 10.1007/978-1-4419-0298-6_5 19731621

[B18] D’Agostino M-A Said-Nahal R Hacquard-Bouder C Brasseur J-L Dougados M Breban M Assessment of peripheral enthesitis in the spondylarthropathies by ultrasonography combined with power doppler: a cross-sectional study Arthritis & Rheumatism 2003 14 2 523 533 10.1002/art.10812 12571863

[B19] McGonagle D Gibbon W O’Connor P Green M Pease C Emery P Characteristic magnetic resonance imaging entheseal changes of knee synovitis in spondylarthropathy Arthritis & Rheumatism 1998 14 4 694 700 10.1002/1529-0131(199804)41:4<694::AID-ART17>3.0.CO;2-# 9550479

[B20] Siegle I Klein T Backman JT Saal JG Nüsing RM Fritz P Expression of cyclooxygenase 1 and cyclooxygenase 2 in human synovial tissue: Differential elevation of cyclooxygenase 2 in inflammatory joint diseases Arthritis & Rheumatism 1998 14 1 122 129 10.1002/1529-0131(199801)41:1<122::AID-ART15>3.0.CO;2-8 9433877

[B21] Poddubnyy D Rudwaleit M Haibel H Listing J Märker-Hermann E Zeidler H Braun J Sieper J Effect of non-steroidal anti-inflammatory drugs on radiographic spinal progression in patients with axial spondyloarthritis: results from the German Spondyloarthritis Inception Cohort Ann Rheum Dis 2012 In Press 10.1136/annrheumdis-2011-201252 22459541

[B22] Kroon F Landewé R Dougados M van der Heijde D Continuous NSAID use reverts the effects of inflammation on radiographic progression in patients with ankylosing spondylitis Ann Rheum Dis 2012 In Press 10.1136/annrheumdis-2012-201370 22532639

[B23] Wanders A Heijde D Landewe R Behier JM Calin A Olivieri I Zeidler H Dougados M Nonsteroidal antiinflammatory drugs reduce radiographic progression in patients with ankylosing spondylitis: a randomized clinical trial Arthritis Rheum 2005 14 6 1756 1765 10.1002/art.21054 15934081

[B24] Evans DM Spencer CC Pointon JJ Su Z Harvey D Kochan G Opperman U Dilthey A Pirinen M Stone MA Interaction between ERAP1 and HLA-B27 in ankylosing spondylitis implicates peptide handling in the mechanism for HLA-B27 in disease susceptibility Nat Genet 2011 14 8 761 767 10.1038/ng.873 21743469 PMC3640413

[B25] Zhang J Wang JHC Production of PGE2 increases in tendons subjected to repetitive mechanical loading and induces differentiation of tendon stem cells into non-tenocytes J Orthop Res 2010 14 2 198 203 19688869 10.1002/jor.20962

[B26] Chen Q Muramoto K Masaaki N Ding Y Yang H Mackey M Li W Inoue Y Ackermann K Shirota H A novel antagonist of the prostaglandin E2 EP4 receptor inhibits Th1 differentiation and Th17 expansion and is orally active in arthritis models Br J Pharmacol 2010 14 2 292 310 10.1111/j.1476-5381.2010.00647.x 20423341 PMC2874852

[B27] Chen C-H Lin K-C Yu DTY Yang C Huang F Chen H-A Liang T-H Liao H-T Tsai C-Y Wei JCC Serum matrix metalloproteinases and tissue inhibitors of metalloproteinases in ankylosing spondylitis: MMP-3 is a reproducibly sensitive and specific biomarker of disease activity Rheumatology 2006 14 4 414 420 10.1093/rheumatology/kei208 16287916

[B28] Soliman E Labib W El-tantawi G Hamimy A Alhadidy A Aldawoudy A Role of matrix metalloproteinase-3 (MMP-3) and magnetic resonance imaging of sacroiliitis in assessing disease activity in ankylosing spondylitis Rheumatol Int 2011 14 6 1 10 21431945 10.1007/s00296-011-1852-8

[B29] Kruithof E De Rycke L Vandooren B De Keyser F FitzGerald O McInnes I Tak PP Bresnihan B Veys EM Baeten D Identification of synovial biomarkers of response to experimental treatment in early-phase clinical trials in spondylarthritis Arthritis Rheum 2006 14 6 1795 1804 10.1002/art.21914 16729282

[B30] Yang C Gu J Rihl M Baeten D Huang F Zhao M Zhang H Maksymowych WP De Keyser F Veys EM Serum levels of matrix metalloproteinase 3 and macrophage colony-stimulating factor 1 correlate with disease activity in ankylosing spondylitis Arthritis Care Res 2004 14 5 691 699 10.1002/art.20696 15478146

[B31] Vandooren B Kruithof E Yu DTY Rihl M Gu J De Rycke L Van Den Bosch F Veys EM De Keyser F Baeten D Involvement of matrix metalloproteinases and their inhibitors in peripheral synovitis and down-regulation by tumor necrosis factor α blockade in spondylarthropathy Arthritis & Rheumatism 2004 14 9 2942 2953 10.1002/art.20477 15457463

[B32] Appel H Ruiz-Heiland G Listing J Zwerina J Herrmann M Mueller R Haibel H Baraliakos X Hempfing A Rudwaleit M Altered skeletal expression of sclerostin and its link to radiographic progression in ankylosing spondylitis Arthritis Rheum 2009 14 11 3257 3262 10.1002/art.24888 19877044

[B33] Haynes K Pettit A Duan R Tseng H-W Glant T Brown M Thomas G Excessive bone formation in a mouse model of ankylosing spondylitis is associated with decreases in Wnt pathway inhibitors Arthritis Res Ther 2012 14 6 R253 10.1186/ar4096 23171658 PMC3674607

