# Supplementary material for: Expression profiling in spondyloarthropathy synovial biopsies highlights changes in expression of inflammatory genes in conjunction with tissue remodelling genes
Source: BMC Musculoskelet Disord. 2013 Dec 15;14:354. doi: 10.1186/1471-2474-14-354 (PMC3878669; doi:10.1186/1471-2474-14-354)
Supplement: Additional file 1: Table S1 — Clinical data for patients and controls. [file 1471-2474-14-354-S1.docx]

Supplementary Table 1. Clinical data for patients and controls.

| **Sex** | **Age** | **Disease.** | **CRP** | **HLAB27** | **Swollen joint count** | **Tender Joint Count** |
| --- | --- | --- | --- | --- | --- | --- |
| M | 50 | AS | 22 | Positive | 2 | 4 |
| M | 75 | AS | 41 | Positive | 2 | 2 |
| M | 47 | SpA | 12 | Positive | 2 | 4 |
| F | 19 | SpA | 126 | ND | 2 | 4 |
| F | 65 | SpA | 4 | ND | 1 | 4 |
| M | 75 | SpA | 94 | Positive | 1 | 3 |
| M | 74 | SpA | 43 | Positive | 1 | 15 |
| M | 74 | SpA | 5 | Positive | 12 | 20 |
| M | 36 | Normal | ND | ND | ND | ND |
| F | 23 | Normal | ND | ND | ND | ND |
| M | 47 | Normal | ND | ND | ND | ND |
| F | 25 | Normal | ND | ND | ND | ND |
| M | 42 | Normal | ND | ND | ND | ND |
| F | 55 | Normal | ND | ND | ND | ND |
| M | 35 | Normal | ND | ND | ND | ND |
| M | 40 | Normal | ND | ND | ND | ND |
| M | 50 | OA | ND | ND | ND | ND |
| F | 69 | OA | ND | ND | ND | ND |
| M | 72 | OA | ND | ND | ND | ND |
| F | 76 | RA | 109 | ND | ND | ND |
| M | 58 | RA | 49 | ND | 24 | 27 |
| F | 57 | RA | ND | ND | ND | ND |
| F | 49 | RA | 3 | ND | ND | ND |
| M | 71 | RA | 66 | ND | 15 | 15 |
| F | 77 | RA | ND | ND | ND | ND |
| F | 71 | RA | 9 | ND | ND | ND |
| F | 56 | RA | 167 | ND | 19 | 26 |
| F | 59 | RA | 82 | ND | 24 | 26 |
| F | 75 | RA | 51 | ND | 14 | 22 |
| M | 59 | RA | ND | ND | 15 | 22 |
| M | 72 | RA | 27 | ND | 18 | 23 |
| F | 74 | RA | 26 | ND | 13 | 22 |
| M | 77 | RA | 16 | ND | 12 | 16 |
| F | 75 | RA | 15 | ND | 10 | 12 |
| F | 60 | RA | 80 | ND | 3 | 5 |
| M | 70 | RA | 116 | ND | 15 | 12 |

AS = Ankylosing spondylitis, SpA = seronegative spondyloarthritis, RA = rheumatoid arthritis

CRP = C-reactive protein – a serum assay of inflammation

HLAB27 – Indicates whether a subject carries the HLAB27 allele

ND = Not done
