# Supplementary material for: Expression profiling in spondyloarthropathy synovial biopsies highlights changes in expression of inflammatory genes in conjunction with tissue remodelling genes
Source: BMC Musculoskelet Disord. 2013 Dec 15;14:354. doi: 10.1186/1471-2474-14-354 (PMC3878669; doi:10.1186/1471-2474-14-354)
Supplement: Additional file 2: Table S2 — Class comparison of the Norm-OA and AS-SpA groups identified 416 differentially expressed genes (p < 0.01) ranging from a 4.7-fold up-regulation to a 4.6-fold down-regulation. [file 1471-2474-14-354-S2.doc]

Supplementary Table 2. Class comparison of the Norm-OA and AS-SpA groups identified 416 differentially expressed genes (p<0.01) ranging from a 4.7-fold up-regulation to a 4.6-fold down-regulation.

| **Parametric p-value** | **Geometric mean of intensities in Norm-OA** | **Geometric mean of intensities in AS-SpA** | **Fold-change** | **Gene symbol** |
| --- | --- | --- | --- | --- |
| 0.0052552 | 480.66 | 2260.32 | 4.70 | MMP3 |
| 0.0028544 | 601.53 | 2391.11 | 3.98 | CD300LF |
| 0.0012548 | 404.29 | 1543.71 | 3.82 | CCL19 |
| 0.0010531 | 199.47 | 701.47 | 3.52 | MMP1 |
| 0.0090337 | 349.07 | 1135.25 | 3.25 | ITGAL |
| 0.0061944 | 373.92 | 1042.64 | 2.79 | LTB |
| 0.0087603 | 326.74 | 847.24 | 2.59 | VEGFC |
| 0.004464 | 346.36 | 883.94 | 2.55 | TRIM17 |
| 0.0033557 | 451.76 | 1150.34 | 2.55 | VDR |
| 0.0044885 | 186.99 | 453.35 | 2.42 | CHI3L2 |
| 0.0021331 | 172.16 | 412.32 | 2.40 | ALPK2 |
| 0.0089321 | 468.51 | 1114.37 | 2.38 | LTA |
| 0.0039561 | 282.19 | 664.05 | 2.35 | SIGLEC10 |
| 0.0034623 | 388.75 | 894.53 | 2.30 | C14orf131 |
| 0.0033679 | 1317.35 | 3019.92 | 2.29 | ALPL |
| 0.0049438 | 1718.82 | 3863.28 | 2.25 | TMEM49 |
| 0.003497 | 572.80 | 1272.76 | 2.22 | SUSD1 |
| 0.0084763 | 184.52 | 400.25 | 2.17 | IL1B |
| 0.0095449 | 498.42 | 1066.65 | 2.14 | CD180 |
| 0.0005348 | 195.88 | 412.55 | 2.11 | TMEM163 |
| 0.0096727 | 361.55 | 756.65 | 2.09 | CCT3 |
| 0.0049458 | 179.43 | 374.64 | 2.09 | PCBP3 |
| 0.0043728 | 3045.69 | 6358.81 | 2.09 | BRSK1 |
| 0.0063204 | 400.06 | 813.45 | 2.03 | ARHGAP30 |
| 0.000176 | 407.92 | 829.29 | 2.03 | PLTP |
| 0.0030766 | 228.85 | 460.91 | 2.01 | PTGDS |
| 0.0062872 | 211.02 | 423.68 | 2.01 | PDIA4 |
| 0.0092742 | 1282.83 | 2523.95 | 1.97 | IRF5 |
| 0.0046862 | 263.87 | 514.34 | 1.95 | FYB |
| 0.0007994 | 177.10 | 336.64 | 1.90 | C20orf165 |
| 0.001784 | 305.14 | 571.60 | 1.87 | KYNU |
| 0.0023969 | 966.91 | 1809.80 | 1.87 | GALNAC4S-6ST |
| 0.0039523 | 1106.01 | 2047.62 | 1.85 | IFI30 |
| 0.0090549 | 323.27 | 591.36 | 1.83 | B4GALT3 |
| 0.006714 | 202.38 | 369.61 | 1.83 | MYO1F |
| 0.0014196 | 243.40 | 444.45 | 1.83 | TAS1R1 |
| 0.0090451 | 492.15 | 897.33 | 1.82 | FAM20A |
| 0.0056326 | 361.02 | 657.73 | 1.82 | CAPN12 |
| 0.0051321 | 571.66 | 1039.82 | 1.82 | GMPPA |
| 0.0033487 | 236.95 | 430.82 | 1.82 | VNN2 |
| 0.0040171 | 270.38 | 482.28 | 1.78 | LYNX1 |
| 0.0032275 | 2076.99 | 3700.03 | 1.78 | SLC2A6 |
| 0.0036045 | 355.49 | 632.02 | 1.78 | LAT2 |
| 0.0078616 | 189.31 | 336.53 | 1.78 | TSPAN32 |
| 0.0018001 | 386.54 | 686.05 | 1.77 | FGF10 |
| 0.0090133 | 1178.22 | 2082.77 | 1.77 | SLCO2B1 |
| 0.0082402 | 495.21 | 838.08 | 1.69 | TWISTNB |
| 0.0091621 | 198.82 | 333.60 | 1.68 | SAMD3 |
| 0.0023233 | 183.07 | 306.74 | 1.68 | ADCY1 |
| 0.0052168 | 237.51 | 396.40 | 1.67 | C1GALT1 |
| 0.0065414 | 1878.89 | 3108.03 | 1.65 | NEK6 |
| 0.0027512 | 970.96 | 1599.19 | 1.65 | ANKMY1 |
| **0.0091533** | **169.46** | **279.07** | **1.65** | **FCGR1A** |
| 0.0061764 | 578.98 | 953.17 | 1.65 | ACOT11 |
| 0.001587 | 513.81 | 844.88 | 1.64 | CMTM7 |
| 0.0015067 | 404.43 | 664.98 | 1.64 | OBFC2A |
| 0.0049875 | 227.33 | 369.49 | 1.63 | PDE4B |
| 0.0081153 | 243.01 | 393.93 | 1.62 | KLF16 |
| 0.009574 | 1301.64 | 2102.07 | 1.61 | C9orf142 |
| 0.0004517 | 1626.74 | 2593.87 | 1.59 | WDR4 |
| 0.00733 | 248.16 | 395.26 | 1.59 | ERP29 |
| 0.0068185 | 170.76 | 271.83 | 1.59 | BATF2 |
| 0.0054097 | 470.09 | 725.84 | 1.54 | BTN3A2 |
| 0.0084555 | 1165.78 | 1777.39 | 1.52 | MTG1 |
| 0.0063248 | 557.24 | 847.69 | 1.52 | RANGAP1 |
| 0.0052022 | 198.07 | 297.24 | 1.50 | LOC340602 |
| 0.009398 | 227.17 | 340.48 | 1.50 | APOC1 |
| 0.0049524 | 408.32 | 611.41 | 1.50 | DCBLD1 |
| 0.0048931 | 322.28 | 482.30 | 1.50 | ARHGAP9 |
| 0.0011088 | 281.83 | 416.15 | 1.48 | ASCL2 |
| 0.0074858 | 374.71 | 545.65 | 1.46 | PFTK1 |
| 0.0092174 | 1693.69 | 2453.95 | 1.45 | C7orf54 |
| **0.0037122** | **1056.67** | **1521.71** | **1.44** | **CD40** |
| 0.0007618 | 445.86 | 641.24 | 1.44 | ZNF655 |
| 0.0052509 | 231.08 | 332.04 | 1.44 | MTP18 |
| 0.0023957 | 1783.79 | 2554.28 | 1.43 | MZF1 |
| 0.0026401 | 675.93 | 967.46 | 1.43 | PRMT1 |
| 0.0049056 | 234.95 | 334.62 | 1.42 | LTA4H |
| 0.0091205 | 160.99 | 229.22 | 1.42 | SAA2 |
| 0.0024623 | 184.34 | 262.21 | 1.42 | CCL8 |
| 0.0057398 | 460.83 | 653.66 | 1.42 | CHD1L |
| 0.0022428 | 212.15 | 300.71 | 1.42 | SRFBP1 |
| 0.0037754 | 702.67 | 995.40 | 1.42 | HLA-DRB6 |
| 0.0038457 | 4802.28 | 6796.02 | 1.42 | ECGF1 |
| 0.0060739 | 449.44 | 635.75 | 1.41 | PSMB8 |
| 0.0042307 | 1140.49 | 1611.37 | 1.41 | SHMT2 |
| 0.0044613 | 1136.14 | 1599.41 | 1.41 | KRT10 |
| 0.0011415 | 176.11 | 247.40 | 1.40 | EMR2 |
| 0.0074564 | 254.14 | 355.42 | 1.40 | SLC4A8 |
| 0.0004914 | 2062.74 | 2868.06 | 1.39 | EIF2AK4 |
| **0.000779** | **172.39** | **239.19** | **1.39** | **CLEC12A** |
| 0.008019 | 446.23 | 618.84 | 1.39 | TYW1 |
| 0.0087593 | 1057.45 | 1463.96 | 1.38 | CCDC72 |
| 0.0012179 | 483.44 | 668.05 | 1.38 | TTC3 |
| 0.0062513 | 151.98 | 209.72 | 1.38 | LSR |
| 0.009768 | 521.95 | 717.63 | 1.37 | PRDX5 |
| 0.0095253 | 402.32 | 550.64 | 1.37 | ATR |
| 0.0006015 | 160.58 | 218.90 | 1.36 | C11orf75 |
| 0.0004984 | 308.89 | 418.07 | 1.35 | SON |
| 0.0047324 | 180.97 | 244.48 | 1.35 | RAB15 |
| 0.0019869 | 203.48 | 274.65 | 1.35 | DGKQ |
| 0.0015981 | 440.52 | 594.25 | 1.35 | SNHG5 |
| 0.0035196 | 260.62 | 351.02 | 1.35 | KSR1 |
| 0.0013944 | 298.53 | 400.23 | 1.34 | CASP8 |
| 0.0070139 | 230.06 | 307.38 | 1.34 | SEC22B |
| 0.0004248 | 485.44 | 647.65 | 1.33 | PRPF4 |
| 0.0055436 | 526.83 | 699.27 | 1.33 | ABI3 |
| 0.0006097 | 240.41 | 318.81 | 1.33 | PSMA3 |
| 0.0095759 | 701.26 | 925.57 | 1.32 | ETNK1 |
| 0.0021776 | 2995.94 | 3913.46 | 1.31 | SIPA1 |
| 0.0027396 | 276.38 | 360.36 | 1.30 | LINS1 |
| 0.0063717 | 245.97 | 320.58 | 1.30 | JOSD3 |
| 0.0016232 | 558.21 | 727.19 | 1.30 | THTPA |
| 0.0027402 | 402.74 | 523.76 | 1.30 | MRPS26 |
| 0.0065223 | 264.97 | 344.08 | 1.30 | UCRC |
| 0.0018713 | 354.05 | 459.60 | 1.30 | C4orf33 |
| 0.0026926 | 162.57 | 210.21 | 1.29 | GPATCH4 |
| 0.0094392 | 250.62 | 323.22 | 1.29 | C5orf33 |
| 0.0069653 | 222.88 | 287.27 | 1.29 | NID2 |
| 0.0089504 | 1464.25 | 1884.27 | 1.29 | TMEM103 |
| 0.0002288 | 467.65 | 600.02 | 1.28 | OPA3 |
| 0.0048064 | 234.39 | 299.83 | 1.28 | ZCCHC11 |
| 0.0055375 | 199.57 | 254.69 | 1.28 | ZNF136 |
| 0.0093889 | 290.72 | 369.36 | 1.27 | PPIB |
| 0.0028017 | 178.04 | 225.95 | 1.27 | ZNF7 |
| 0.0074378 | 277.19 | 350.12 | 1.26 | SKAP2 |
| 0.009857 | 773.32 | 975.15 | 1.26 | GPC6 |
| 0.0066473 | 232.97 | 293.41 | 1.26 | FUS |
| 0.0068133 | 251.94 | 317.30 | 1.26 | DDX55 |
| 0.0071161 | 202.94 | 255.10 | 1.26 | MPHOSPH6 |
| 0.0016344 | 172.30 | 216.35 | 1.26 | KCNQ3 |
| 0.0051091 | 163.63 | 205.16 | 1.25 | RRP9 |
| 0.0010743 | 212.06 | 265.29 | 1.25 | HIF1A |
| 0.004985 | 1385.14 | 1722.78 | 1.24 | FAM111A |
| 0.0012011 | 215.04 | 266.23 | 1.24 | PSMC4 |
| 0.0023794 | 4958.87 | 6108.04 | 1.23 | TIMP1 |
| 0.0052203 | 181.49 | 222.69 | 1.23 | FAM71F1 |
| 0.0024792 | 199.48 | 244.01 | 1.22 | KDELC1 |
| 0.0033482 | 181.53 | 221.55 | 1.22 | ZNF780B |
| 0.0024567 | 223.86 | 272.73 | 1.22 | NMI |
| 0.0057027 | 355.23 | 432.71 | 1.22 | SIRPA |
| 0.0051549 | 798.59 | 967.70 | 1.21 | KIAA0152 |
| 0.0099219 | 195.20 | 235.39 | 1.21 | SRGAP2 |
| 0.0078361 | 1794.12 | 2156.01 | 1.20 | NISCH |
| 0.0046415 | 168.47 | 202.41 | 1.20 | IGSF6 |
| 0.0070451 | 191.11 | 228.11 | 1.19 | BTN3A1 |
| 0.0044174 | 313.13 | 370.62 | 1.18 | NARG1L |
| 0.004828 | 189.17 | 223.41 | 1.18 | ARHGAP22 |
| 0.0087822 | 3623.39 | 4257.54 | 1.18 | CENTA1 |
| 0.0054467 | 166.69 | 194.98 | 1.17 | PTX3 |
| 0.0016106 | 164.91 | 191.85 | 1.16 | INTS7 |
| 0.0085092 | 275.29 | 316.42 | 1.15 | TRIM5 |
| 0.0062096 | 226.85 | 260.23 | 1.15 | GOPC |
| 0.0095104 | 160.71 | 180.01 | 1.12 | POLD3 |
| 0.0037198 | 156.25 | 167.86 | 1.07 | ZBTB8OS |
| 0.0027865 | 158.25 | 169.47 | 1.07 | RAPH1 |
| 0.0010044 | 149.60 | 158.61 | 1.06 | CDK5R1 |
| 0.0025666 | 153.08 | 161.84 | 1.06 | EPS8L1 |
| 0.0068465 | 150.79 | 154.05 | 1.02 | MAGEE2 |
| 0.009343 | 154.93 | 151.10 | -1.03 | UGT1A6 |
| 0.0016061 | 153.91 | 147.94 | -1.04 | GABRG3 |
| 0.0052845 | 167.42 | 152.71 | -1.10 | LGI1 |
| 0.0027987 | 177.53 | 157.92 | -1.12 | RDH10 |
| 0.0082883 | 3590.74 | 3050.24 | -1.18 | RAC1 |
| 0.0078655 | 282.11 | 232.70 | -1.21 | CCL28 |
| 0.00619 | 256.27 | 210.41 | -1.22 | LOC221091 |
| 0.0099569 | 646.06 | 525.90 | -1.23 | RAB11FIP2 |
| 0.0088848 | 201.48 | 163.22 | -1.23 | BDH1 |
| 0.0006959 | 195.12 | 158.05 | -1.23 | MYOM2 |
| 0.0056615 | 4847.12 | 3922.84 | -1.24 | ZNF385A |
| 0.0036357 | 499.03 | 403.15 | -1.24 | FRS2 |
| 0.0049319 | 4955.36 | 3962.92 | -1.25 | SH3KBP1 |
| 0.0005456 | 2170.81 | 1726.49 | -1.26 | LGMN |
| 0.0021498 | 363.88 | 289.14 | -1.26 | PPIA |
| 0.0051334 | 206.86 | 163.91 | -1.26 | NMT2 |
| 0.0067067 | 630.52 | 496.10 | -1.27 | ITGB1BP1 |
| 0.0086339 | 1306.05 | 1027.08 | -1.27 | C19orf70 |
| 0.0051795 | 217.99 | 171.18 | -1.27 | FNDC1 |
| 0.0041926 | 1633.07 | 1282.32 | -1.27 | PDCL3 |
| 0.0008365 | 1612.86 | 1265.39 | -1.27 | NF1 |
| 0.0086488 | 1443.27 | 1132.31 | -1.27 | SLC35B2 |
| 0.0091983 | 301.76 | 236.67 | -1.28 | HN1L |
| 0.0076757 | 211.74 | 165.37 | -1.28 | MIPOL1 |
| 0.0068933 | 241.67 | 187.61 | -1.29 | CD34 |
| 0.0031269 | 361.66 | 279.95 | -1.29 | BMPR2 |
| 0.0083648 | 2331.86 | 1803.43 | -1.29 | UTRN |
| 0.0071957 | 2519.07 | 1938.02 | -1.30 | MKLN1 |
| 0.0007978 | 543.90 | 416.11 | -1.31 | DHX40 |
| 0.002076 | 1181.52 | 902.93 | -1.31 | PLEKHA3 |
| 0.001666 | 1595.28 | 1217.91 | -1.31 | CASC3 |
| 0.0022087 | 3495.95 | 2668.56 | -1.31 | GHITM |
| 0.005268 | 1045.90 | 795.89 | -1.31 | ZCCHC2 |
| 0.00044 | 240.22 | 182.11 | -1.32 | AXL |
| 0.0062411 | 478.69 | 361.48 | -1.32 | RUFY2 |
| 0.0085272 | 1937.01 | 1440.20 | -1.34 | CRIM1 |
| 0.0051297 | 216.93 | 161.25 | -1.35 | PRRG3 |
| 0.0096861 | 233.35 | 173.42 | -1.35 | COL8A1 |
| 0.0021166 | 923.83 | 685.59 | -1.35 | STS |
| 0.0049396 | 6004.80 | 4449.41 | -1.35 | WISP2 |
| 0.008714 | 282.55 | 208.65 | -1.35 | TESK2 |
| 0.0022033 | 232.99 | 172.00 | -1.35 | P2RY14 |
| 0.0032473 | 442.96 | 326.48 | -1.36 | LOC116236 |
| 0.007946 | 2312.59 | 1690.22 | -1.37 | PTPRM |
| 0.0023242 | 1668.20 | 1211.26 | -1.38 | ZNF25 |
| 0.0036643 | 3766.50 | 2718.20 | -1.39 | DCN |
| 0.0053805 | 252.13 | 181.92 | -1.39 | MREG |
| 0.0058775 | 688.70 | 492.55 | -1.40 | RAB34 |
| 0.0090619 | 288.19 | 204.65 | -1.41 | DMD |
| 0.0071422 | 555.05 | 392.12 | -1.42 | RBMS3 |
| 0.0023315 | 255.62 | 180.50 | -1.42 | NOV |
| 0.0053122 | 4863.53 | 3430.54 | -1.42 | CRAT |
| 0.0025079 | 3207.87 | 2254.10 | -1.42 | SLC10A3 |
| 0.0071974 | 1539.61 | 1073.37 | -1.43 | CUGBP2 |
| 0.0023391 | 460.29 | 320.54 | -1.44 | PIGC |
| 0.0034185 | 1977.15 | 1376.56 | -1.44 | ANO10 |
| 0.0092573 | 14229.42 | 9899.15 | -1.44 | C10orf116 |
| 0.0032752 | 254.97 | 176.95 | -1.44 | LAMA3 |
| 0.0077534 | 683.58 | 472.76 | -1.45 | RBM15B |
| 0.0089811 | 2100.72 | 1449.82 | -1.45 | CSRP1 |
| 0.0064125 | 611.46 | 421.91 | -1.45 | UCHL1 |
| 0.0042853 | 313.23 | 215.90 | -1.45 | SYNM |
| 0.0067914 | 333.28 | 229.58 | -1.45 | KCNF1 |
| 0.0065071 | 636.03 | 435.28 | -1.46 | TRMT2B |
| 0.001045 | 7965.44 | 5446.23 | -1.46 | DCN |
| 0.0058486 | 2617.78 | 1788.65 | -1.46 | SF1 |
| 0.0064666 | 988.68 | 674.93 | -1.46 | KREMEN1 |
| 0.0002191 | 518.90 | 353.68 | -1.47 | RERE |
| 0.0060518 | 248.13 | 169.02 | -1.47 | RICS |
| 0.0068638 | 4512.11 | 3071.22 | -1.47 | GNG12 |
| 0.0010297 | 842.72 | 572.88 | -1.47 | CCNDBP1 |
| 0.003808 | 605.03 | 410.84 | -1.47 | STX2 |
| 0.001791 | 2254.45 | 1524.48 | -1.48 | PEPD |
| 0.0061959 | 337.16 | 225.58 | -1.49 | NMB |
| 0.0022849 | 2391.27 | 1594.68 | -1.50 | RRAS |
| 0.0077634 | 320.54 | 213.50 | -1.50 | KCNAB1 |
| 0.0002584 | 1575.58 | 1032.29 | -1.53 | NFIC |
| 0.0039901 | 417.43 | 272.67 | -1.53 | HPS6 |
| 0.0094822 | 287.61 | 187.69 | -1.53 | MASP1 |
| 0.0014007 | 297.86 | 192.67 | -1.55 | PPL |
| 9.90E-05 | 3150.59 | 2036.12 | -1.55 | VIM |
| 0.0088851 | 604.22 | 390.47 | -1.55 | THEM2 |
| 0.001494 | 302.06 | 195.19 | -1.55 | PLA2R1 |
| 0.009791 | 3515.12 | 2268.16 | -1.55 | LTBP4 |
| 0.0070273 | 1724.19 | 1110.88 | -1.55 | CDC42EP4 |
| 0.0036289 | 433.50 | 279.15 | -1.55 | PDGFB |
| 0.0093471 | 1816.29 | 1168.44 | -1.55 | MTMR2 |
| 0.0035978 | 609.20 | 390.15 | -1.56 | ATP2B4 |
| 0.0079965 | 2017.93 | 1282.48 | -1.57 | SRC |
| 0.0028861 | 1000.06 | 635.31 | -1.57 | ZNF219 |
| 0.0070659 | 1057.75 | 670.91 | -1.58 | C14orf49 |
| 0.0096891 | 2629.30 | 1661.54 | -1.58 | FILIP1L |
| 0.0037255 | 868.19 | 548.37 | -1.58 | C14orf139 |
| 0.000955 | 302.69 | 191.02 | -1.58 | RGS6 |
| 0.0085477 | 487.59 | 307.64 | -1.58 | FHL1 |
| 0.0085149 | 507.65 | 320.02 | -1.59 | LATS2 |
| 0.0009656 | 1316.07 | 829.43 | -1.59 | Sep-10 |
| 0.0097961 | 706.12 | 444.65 | -1.59 | SORT1 |
| 0.008123 | 653.16 | 411.19 | -1.59 | TMEM98 |
| 0.0063523 | 273.67 | 172.16 | -1.59 | PCDHGA4 |
| 0.0081567 | 1714.07 | 1078.33 | -1.59 | SLC37A3 |
| 0.0042673 | 2672.18 | 1675.06 | -1.60 | MGLL |
| 0.0057168 | 1048.13 | 656.85 | -1.60 | RBMS3 |
| 0.0075123 | 436.82 | 272.90 | -1.60 | MAPK1 |
| 0.0087239 | 1875.06 | 1169.60 | -1.60 | PKN3 |
| 0.00867 | 550.56 | 343.40 | -1.60 | PHLDA3 |
| 0.0001526 | 797.46 | 496.01 | -1.61 | YPEL1 |
| 0.0046935 | 1013.77 | 630.44 | -1.61 | GRLF1 |
| 0.0059038 | 1818.66 | 1130.89 | -1.61 | SPTBN1 |
| 0.0018477 | 1801.21 | 1117.95 | -1.61 | PLAGL1 |
| 0.0083947 | 1354.50 | 838.91 | -1.61 | SKI |
| 0.0010034 | 3152.18 | 1951.96 | -1.61 | CD36 |
| 0.0016179 | 1014.12 | 627.72 | -1.62 | SNX21 |
| 0.0078319 | 255.08 | 157.65 | -1.62 | CCDC74B |
| 0.0099389 | 595.12 | 367.58 | -1.62 | TRIO |
| 0.0080613 | 847.72 | 522.60 | -1.62 | MOCS2 |
| 0.0082415 | 336.93 | 206.91 | -1.63 | SLC13A4 |
| 0.0021516 | 352.75 | 216.61 | -1.63 | PIK3R1 |
| 0.0098459 | 643.24 | 394.34 | -1.63 | EGFR |
| 0.0002887 | 299.52 | 181.18 | -1.65 | DMD |
| 0.000121 | 352.29 | 212.72 | -1.66 | C9orf61 |
| 0.0037852 | 1017.82 | 613.80 | -1.66 | ZMAT1 |
| 0.0089626 | 251.53 | 151.65 | -1.66 | TTLL10 |
| 0.0063771 | 1061.87 | 637.22 | -1.67 | C17orf58 |
| 0.0068285 | 3433.71 | 2051.49 | -1.67 | MUSTN1 |
| 0.0085365 | 275.96 | 164.35 | -1.68 | CORO2B |
| 0.0070451 | 513.85 | 305.24 | -1.68 | GLRX2 |
| 0.0075816 | 679.96 | 403.27 | -1.69 | EDA |
| 0.0002726 | 341.66 | 202.06 | -1.69 | IRS1 |
| 0.007538 | 826.02 | 488.01 | -1.69 | EDA |
| 0.0056515 | 411.57 | 242.60 | -1.70 | HSF2 |
| 0.0032867 | 455.40 | 268.09 | -1.70 | PALLD |
| 0.0032247 | 282.75 | 166.07 | -1.70 | BEST4 |
| 0.0044663 | 425.34 | 248.38 | -1.71 | ST8SIA1 |
| 0.0018757 | 1023.88 | 597.59 | -1.71 | ITPR1 |
| 0.0068263 | 923.45 | 538.26 | -1.72 | SH3BP4 |
| 0.0013728 | 849.04 | 494.87 | -1.72 | SHMT1 |
| 0.0012353 | 1504.40 | 875.72 | -1.72 | PPP2R3A |
| 0.0041962 | 820.63 | 477.38 | -1.72 | MEOX1 |
| 0.0086215 | 370.76 | 215.57 | -1.72 | FLJ10324 |
| 0.0012574 | 4443.29 | 2579.71 | -1.72 | TLN2 |
| 0.0047206 | 1063.71 | 615.40 | -1.73 | SESTD1 |
| 0.0014859 | 2500.35 | 1445.93 | -1.73 | SOD3 |
| 0.0064434 | 716.59 | 413.37 | -1.73 | GPSM2 |
| 0.0047018 | 886.85 | 511.29 | -1.73 | SNX1 |
| 0.001701 | 290.61 | 167.43 | -1.74 | PKP2 |
| 0.0001319 | 272.59 | 156.90 | -1.74 | C1orf51 |
| 0.0066276 | 315.77 | 181.65 | -1.74 | NANOS1 |
| 0.0012392 | 353.75 | 203.44 | -1.74 | DPP4 |
| 0.0029543 | 4432.30 | 2538.45 | -1.75 | LMOD1 |
| 0.0078049 | 398.32 | 227.98 | -1.75 | SYT1 |
| 0.0026927 | 376.36 | 214.67 | -1.75 | OTUD7A |
| 0.0077809 | 1863.83 | 1060.13 | -1.76 | FILIP1L |
| 0.0045293 | 1284.07 | 728.65 | -1.76 | PFKFB3 |
| 0.0001499 | 2875.88 | 1628.45 | -1.77 | ANXA5 |
| 0.0094148 | 975.15 | 550.11 | -1.77 | SSH3 |
| 0.0048332 | 6188.89 | 3491.19 | -1.77 | PDGFRL |
| 0.0062963 | 1236.41 | 697.31 | -1.77 | EFHD1 |
| 0.0056973 | 458.20 | 257.73 | -1.78 | MAP1B |
| 0.0050988 | 1887.37 | 1058.65 | -1.78 | NOVA1 |
| 0.0009686 | 389.41 | 216.41 | -1.80 | C3orf35 |
| 0.0048678 | 2211.84 | 1221.60 | -1.81 | KCNMA1 |
| 0.0022334 | 2051.21 | 1127.49 | -1.82 | LGR4 |
| 0.0066907 | 2055.51 | 1129.40 | -1.82 | PCOLCE2 |
| 0.0086743 | 329.71 | 181.05 | -1.82 | MMP16 |
| 0.0079874 | 492.66 | 269.79 | -1.83 | C5orf4 |
| 0.0096991 | 596.15 | 325.62 | -1.83 | DBN1 |
| 0.0004808 | 499.98 | 272.85 | -1.83 | ACCN3 |
| 0.0098023 | 890.73 | 484.84 | -1.84 | SCRN1 |
| 0.0076307 | 877.77 | 475.66 | -1.85 | MXRA7 |
| 0.0034633 | 583.15 | 313.88 | -1.86 | C19orf12 |
| 0.0025577 | 532.21 | 281.72 | -1.89 | SH3D19 |
| 0.0024139 | 496.56 | 262.45 | -1.89 | RAB28 |
| 0.0050908 | 676.44 | 356.80 | -1.90 | C19orf12 |
| 0.0014608 | 349.52 | 182.87 | -1.91 | BCAS3 |
| 0.0013373 | 493.98 | 258.39 | -1.91 | SGEF |
| 0.0029718 | 1489.37 | 778.09 | -1.91 | ITIH5 |
| 0.0071277 | 545.62 | 282.95 | -1.93 | OPHN1 |
| 0.0030803 | 426.91 | 221.16 | -1.93 | KCNIP3 |
| 0.0028788 | 1890.93 | 977.48 | -1.93 | LTBP4 |
| 0.0002082 | 1065.08 | 547.83 | -1.94 | CITED4 |
| 0.0089839 | 1641.02 | 843.88 | -1.94 | FLNC |
| 0.0016509 | 553.42 | 283.85 | -1.95 | PDLIM3 |
| 4.98E-05 | 1267.62 | 649.30 | -1.95 | THRA |
| 0.0054402 | 2868.82 | 1465.36 | -1.96 | EGFR |
| 0.0006497 | 1413.50 | 721.45 | -1.96 | OLFML2A |
| 0.0045442 | 441.48 | 224.32 | -1.97 | DBF4B |
| 0.0027854 | 625.29 | 314.33 | -1.99 | MAMDC2 |
| 0.0049643 | 962.62 | 480.16 | -2.00 | LAMA2 |
| 0.0068619 | 373.42 | 185.70 | -2.01 | C1orf165 |
| 0.0041244 | 536.18 | 266.25 | -2.01 | LTBP1 |
| 0.000211 | 521.04 | 256.57 | -2.03 | MSRB3 |
| 0.002202 | 459.46 | 226.23 | -2.03 | CKB |
| 0.0041854 | 1301.81 | 639.39 | -2.04 | MYO5C |
| 0.0007466 | 436.09 | 212.05 | -2.06 | BMP5 |
| 0.0062967 | 371.84 | 180.75 | -2.06 | LMO3 |
| 0.009743 | 1154.00 | 555.20 | -2.08 | SORBS1 |
| 0.0021454 | 523.71 | 250.54 | -2.09 | BIVM |
| 0.0014723 | 422.17 | 201.08 | -2.10 | TPPP |
| 0.0041034 | 414.99 | 196.67 | -2.11 | C11orf41 |
| 0.0061751 | 524.48 | 245.04 | -2.14 | RAB9B |
| 0.0006451 | 1123.67 | 519.85 | -2.16 | C1orf198 |
| 0.0004982 | 979.26 | 443.52 | -2.21 | ZNF704 |
| 0.0070168 | 576.82 | 260.86 | -2.21 | MRPL39 |
| 0.0017668 | 786.12 | 352.06 | -2.23 | STXBP1 |
| 0.0011649 | 652.90 | 292.13 | -2.24 | PHYH |
| 0.0093395 | 1227.21 | 548.13 | -2.24 | CREB3L1 |
| 0.0063064 | 1021.09 | 453.42 | -2.25 | LAMA3 |
| 0.0004427 | 674.48 | 296.67 | -2.27 | ABLIM1 |
| 0.0096803 | 1967.09 | 856.15 | -2.30 | NQO1 |
| 4.36E-05 | 349.87 | 151.02 | -2.32 | MYOC |
| 0.0063601 | 542.16 | 233.22 | -2.32 | FBLN2 |
| 0.0025689 | 650.53 | 276.68 | -2.35 | HSD11B1L |
| 0.0053347 | 1586.00 | 672.98 | -2.36 | WTIP |
| 0.0005106 | 512.68 | 217.44 | -2.36 | ITGA11 |
| 0.0058964 | 579.02 | 245.49 | -2.36 | SHRM |
| 0.0016027 | 466.81 | 197.82 | -2.36 | CACNB1 |
| 0.0037585 | 431.31 | 182.46 | -2.36 | LOC392395 |
| 0.0023696 | 566.04 | 239.41 | -2.36 | CDH13 |
| 0.0086013 | 625.59 | 264.13 | -2.37 | PKD1L2 |
| 0.0083329 | 1215.21 | 512.40 | -2.37 | STXBP6 |
| 0.0052036 | 783.41 | 329.37 | -2.38 | GALNTL2 |
| 0.0027678 | 1696.50 | 708.82 | -2.39 | USP2 |
| 0.0059492 | 514.66 | 214.02 | -2.40 | PGA5 |
| 0.0060933 | 633.49 | 260.52 | -2.43 | SLC35E4 |
| 0.0063288 | 1007.89 | 414.25 | -2.43 | CRABP2 |
| 0.0051863 | 844.12 | 346.92 | -2.43 | CD24 |
| 0.0025767 | 818.70 | 335.42 | -2.44 | C17orf97 |
| 0.0016208 | 589.01 | 237.08 | -2.48 | TSPAN11 |
| 0.001749 | 948.49 | 379.96 | -2.50 | HLF |
| 0.0091082 | 1022.51 | 407.06 | -2.51 | CDO1 |
| 0.0013291 | 700.09 | 277.05 | -2.53 | FBLN2 |
| **0.0036817** | **495.12** | **194.53** | **-2.55** | **TSC22D3** |
| 0.0054985 | 1550.90 | 600.36 | -2.58 | MYOCD |
| 0.0032562 | 465.65 | 178.50 | -2.61 | MAPT |
| 0.0049464 | 681.29 | 261.14 | -2.61 | GPER |
| 0.0002657 | 486.61 | 183.74 | -2.65 | AMPH |
| 0.0097902 | 909.25 | 342.95 | -2.65 | NR1D1 |
| 0.0010818 | 1171.68 | 436.41 | -2.68 | Gcom1 |
| 0.0032632 | 517.49 | 190.98 | -2.71 | DKK3 |
| 0.0010305 | 961.31 | 346.87 | -2.77 | ABLIM1 |
| 0.0043008 | 1197.85 | 423.63 | -2.83 | ITIH5 |
| 0.0016119 | 1267.58 | 424.31 | -2.99 | PTGIS |
| 0.0067585 | 2068.54 | 686.47 | -3.01 | MGST1 |
| 0.0031484 | 970.79 | 311.06 | -3.12 | TNXB |
| 0.0022725 | 771.09 | 239.04 | -3.23 | TSNARE1 |
| 0.0003998 | 751.35 | 230.82 | -3.26 | DUOX1 |
| 0.0075435 | 3111.73 | 946.90 | -3.29 | CCBP2 |
| 0.0047654 | 1096.91 | 332.49 | -3.30 | FGFBP2 |
| 0.0049687 | 1552.60 | 468.75 | -3.31 | CADM1 |
| 0.0046895 | 3714.24 | 1118.57 | -3.32 | CAB39L |
| 0.000777 | 521.19 | 153.81 | -3.39 | CYP4B1 |
| 0.0077863 | 3095.58 | 834.08 | -3.71 | CILP |
| 0.0026437 | 3692.19 | 864.62 | -4.27 | MFAP5 |
| 7.22E-05 | 965.45 | 223.45 | -4.32 | ITGA11 |
| 0.0051548 | 2806.83 | 608.60 | -4.61 | APOD |
| 0.0077124 | 1854.82 | 400.18 | -4.63 | HSPB7 |
